# Inhibitory Effect of Ginkgo Biloba Extract on the Tonus of the Small Intestine and the Colon of Rabbits

**DOI:** 10.3390/molecules15042079

**Published:** 2010-03-24

**Authors:** Vladimir Pilija, Radenkovic Mirjana, Maja Djurendic Brenesel, Mira Popovic, Vesna Ivetic, Svetlana Trivic

**Affiliations:** 1 Clinical Center of Vojvodina, Institute for Forensic Medicine, Hajduk Veljkova 7-9, 21000 Novi Sad, Serbia; 2 Department of Physiology, Medical Faculty, University of Nis, Serbia; 3 Department of Chemistry, Faculty of Sciences, University of Novi Sad, Trg Dositeja Obradovica 3, 21000 Novi Sad, Serbia; 4 Department of Physiology, Medical Faculty, University of Novi Sad, Hajduk Veljkova 3, 21000 Novi Sad, Serbia

**Keywords:** *Ginkgo biloba*, GBE, ileum, colon, acetylcholine, ACh

## Abstract

*Ginkgo biloba* is widely used in folk medicine. Patients very often use the plant preparation with no concern for purity. They also tend to increase the dosage by themselves and this may result in certain insufficiently researched acute effects. Due to this extremely widespread application, the aim of this work is an examination of the possible acute effects of *Ginkgo biloba* on the motility of the small and the large intestine of rabbits. Тhe effects of Gingium**®** - a standardized *ginkgo biloba* extract (GBE) [one milliliter preparation contained 8.8–10.8 mg ginkgo flavonol glycoside and 2.0–2.8 mg lactone ring-containing terpenes (ginkgolides and bilobalides)], on the tonus of isolated segments of the ileum and the colon of rabbits were examined. The experiments were carried out on isolated bowel incisions according to the Magnus method. Data was registered by physiography (Narco-Bio-System). Our results show that GBE (0.006 g/L, - 0.06 g/L) concentration-dependently reduces the tonus of the ileum and the colon of rabbits. Apart from that, GBE reduces the increase of the tonus of the ileum caused by acetylcholine (ACh), but does not change colon tonus intensified by ACh. This indicates that the effects of the used extract in the ileum are predominantly achieved through cholinergic mechanisms, while the relaxant effects in the colon are achieved in some other way.

## Introduction 

*Ginkgo biloba*, the plant from the Ginkgoaceae family, has been used in traditional medicine for more than 2,000 years. Recently it has become fashionable again. *Ginkgo biloba* contains three groups of compounds: ginkgo flavonol glycosides (quercetin, kaempferol, and isorhamnetin), terpenes with lactone rings (ginkgolides A, B, C, J and M and bilobalides) and specific organic acids (ginkgoin and ginkgolin acids). The extract of *Ginkgo biloba* contains the largest percentage of ginkgo flavonol glycosides (22–27%) from the flavonoid group [1,2,3].

*Ginkgo biloba* extract (GBE) dilates the blood vessels and improves tissue vascularity, especially of the brain. Therefore it is widely used in the treatment of ischemic diseases of the brain and the heart [4]. It has been shown that GBE provokes vasodilatation in two ways. It increases the release of NO and through Ca^++^ dependent K^+^ channels it provokes hyperpolarization of smooth muscle cells [5]. In order to achieve good vascularity, the intake must be in several daily dosages [6].

According to some characteristics, the smooth muscles of the digestive tract are similar to vascular smooth muscles (NO is also their major vasodilator), but contrary to them, they are mostly under cholinergic control. Cholinergic control is performed by the neurotransmitter acetylcholine. There are two main classes of ACh receptors: nicotinic ACh receptors and muscarinic ACh receptors. In the digestive canal all types of muscarinic receptors (M1-5) are present, of which the most numerous in the smooth muscles are the receptors of subtype M3.

The active substances from the extract of GBE act primarily through muscarinic receptors and somewhat less through adrenergic receptors. It has been proven experimentally that the application of GBE inhibits the fall of the number of muscarinic and α_2_ adrenergic receptors and at the same time it improves the receipt of choline in synapses and removes toxic free radicals [7,8].

Patients very often use the plant preparation with no care. They tend to increase the dosages by themselves and it may result in certain insufficiently researched acute effects. Due to the extremely widespread application of this substance, the aim of this work is examination of the possible acute effects of GBE on the motility of the small and the large intestine of rabbits.

## Results and Discussion

### The ileum of rabbits

**Figure 1:**
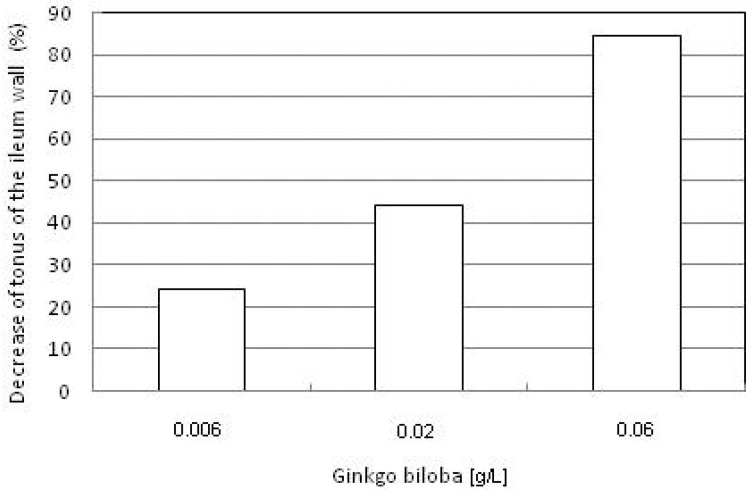
Dosage-dependent decrease of tonus of the ileum wall in the presence of GBE.

GBE in concentrations of 0.006–0.06 g/L causes a dose-dependent decrease of ileum tonus in rabbits (Figure 1). The maximum decrease of tonus was 84.3%. On the basis of the variant in one direction it has been established that there are significant statistical differences between the effects provoked by various concentrations of GBE (F = 63.268; p < 0.01).

**Figure 2:**
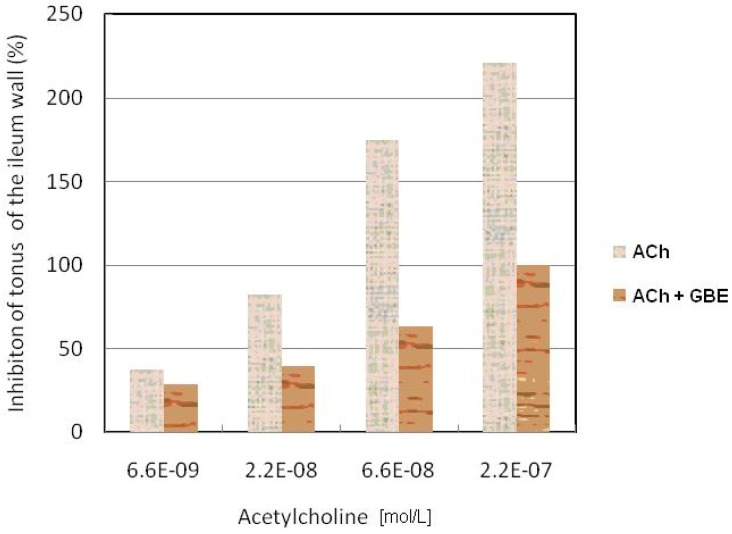
Inhibitory effect of GBE on the increase of tonus of the ileum wall provoked by ACh.

In the control series of the experiment, ACh (6.6 × 10^-9^–2.2 × 10^-7^ mol/L) concentration-dependently increases the ileum tonus of rabbits. The effects of acetylcholine have been presented in Figure 2. In the control series of experiments it has been shown that acetylcholine in the presence of GBE (0.2 g/L) causes a concentration-dependent, but lesser increase of the ileum tonus of rabbits. By Student’s t-test it has been established that there are statistically significant differences (T = 2.836; p < 0.01) between the effect provoked by acetylcholine and ACh in the presence of GBE.

### The colon of rabbit

**Figure 3:**
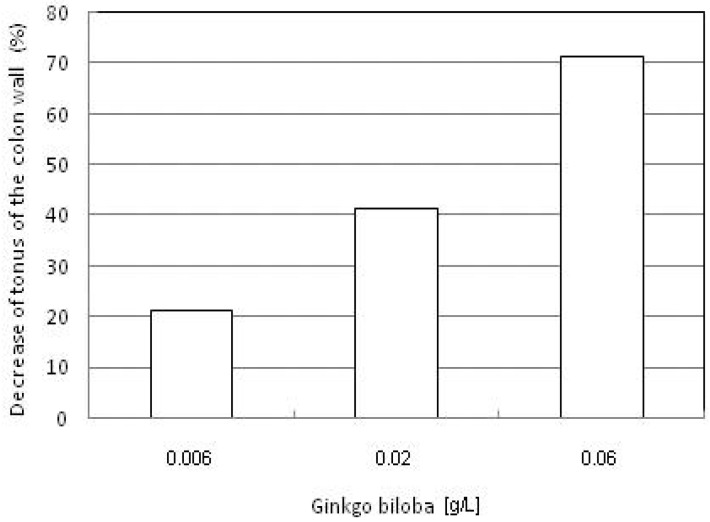
Dosage-dependent decrease of tonus of the colon wall in the presence of GBE.

**Figure 4:**
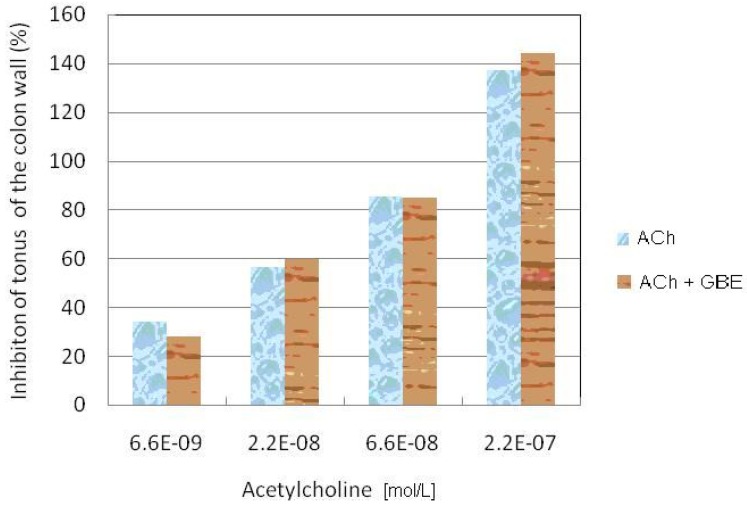
Inhibitory effect of GBE on the increase of tonus of colon wall provoked by Ach.

In concentrations 0.006, 0.02 and 0.06 g/L, GBE causes a decrease of the colon tonus of rabbits (Figure 3). The maximum tonus decrease was 71.09%. According to the variant analysis in one direction it has been established that there are significant statistical differences between the effects caused by various concentrations of GBE (F = 31.363; p < 0.01). ACh (6.6 × 10^-9^–2.2 × 10^-7^ mol/L) causes a concentration-dependent increase in colon tonus (F = 41.137; p > 0.01). The effects of ACh are presented in Figure 4. ACh in the presence of GBE also (0.2 g/L) provokes concentration-dependent increase of colon tonus. GBE does not reduce the effects of ACh. By Student’s t-test it has been established that there are no statistically significant differences (T = 0.157; p > 0.05) between the effects caused by ACh and ACh in the presence of GBE.

Starting from the knowledge that more than 60% of extract is absorbed in the stomach and the small intestine, in our research we monitored the acute effects of GBE effects on pendular movements of the intestinal tract. 

Pendular movements represent spontaneous rhythmical contractions and relaxations of the longitudinal muscular layer that mostly occur by electric activities of the smooth muscle cells themselves. The spontaneous activity is modified by numerous neurotransmitters of the enteric nervous system [9]. This neural plexus that is located in the bowel wall contains a larger number of neurons, and the most common neurotransmitter in it is ACh. Thus, motility and secretion of the gastrointestinal tract are mostly under the influence of cholinergic neurons [10,11]. Our results show that GBE expresses acute effects on the motility of the intestinal tract, strongly reducing the spontaneous activity of both the ileum and the colon. The effects of GBE are concentration-dependent in the examined colon parts. However, GBE does not have the same effect on the tonus of the ileum and the colon intensified by ACh. In the presence of GBE, the effects of ACh in the ileum are reduced and they are unchanged in the colon.

ACh achieves its effects in the digestive tract through nicotine receptors in ganglions and through muscarinic receptors localized in nerve fibers and membranes of smooth muscle cells. According to literature data it is known that in the digestive canals of rabbits muscarinic receptors of types M1, M2 and M3 are present [12]. Their number and sensitivity vary along the digestive canal, and specific blockers of muscarinic receptors express various effects on the spontaneous activity of different bowel parts. On the smooth muscle cells and secretory glands of the gastrointestinal tract the most common are the receptors of M3 subtype [13]. There are various data about receptor subtypes on presynaptic nerve terminals where they carry out auto-regulation of endogenous ACh release. According to some authors these are M2 and according to others M1 receptors [14,15,16,17].

In proportion with the used concentrations, ACh stimulates contractions of the longitudinal muscular layer of the examined bowel parts. In the presence of GBE the effects of ACh are significantly reduced in the ileum and are unchanged in the colon. Such results indicate that in the small intestine GBE displays its effects predominantly through cholinergic path by antagonizing muscarinic receptors and that the mechanism of its effect in the colon is different.

On the basis of our results it is not possible to determine the precise mechanisms of the effect of the extract used. According to literature data, it is possible that the constituents of GBE activate inhibitory presynaptic receptors of M2 or M1 subtype and in this way they provoke tonus fall. On the other hand, it is possible that certain GBE alkaloids block M3 muscarinic receptors on smooth muscle cells of the ileum, decreasing thus ACh effects. Since the population of muscarinic receptors of M3 subtype is the scarcest one in the colon of rabbits, it might be the reason for non-efficiency of GBE on the colon tonus intensified by ACh. It is possible that this extract exerts its effects through NO, which relaxes smooth muscle cells. It may provoke the release of nitric oxide or stimulation of nitric (II) oxide synthetases, but may also effect through adrenergic path or through 5–HT receptors. Possible mechanism GBE modulates the Ca^2+^ influx via L-type *I*_Ca_ channels of smooth muscle cell membrane. Another mechanism is the activation of Ca^2+^-activated K^+^ (*I*_KCa_) channels which leads to hyperpolarization and relaxation [18].

The results obtained show that GBE has a concentration-dependent impact on the motility of the intestinal tract – GBE strongly reduces the spontaneous activity. Although on the basis of our results it is not possible to determine the neurophysiologic mechanisms of effect of GBE, these results are nonetheless very important. 

If the influence of GBE on the digestive tract in humans is like the effects in rabbits then GBE can cause slow passage through the intestine and constipation. If GBA has a concentration-dependent impact in rabbits probably at higher doses (concentration) have more intensive effect in humans also. However, accurate answers to these assumptions require further research.

## Experimental

### Animals

The examinations were carried out on 10 rabbits of Middle European bred chinchilla, of both sexes, aged from 2.5 to 3 months, on body mass of about 3 kg. The rabbits were sacrificed by cervical dislocation and the 2–3 cm-long excisions of ileum or the distal part of the rabbit colon were put into a 20 mL bath for isolated organs, according to the method of Magnus. The bath was filled with Tyrode’s solution for isolated colon (137 mmol NaCl, 2.68 mmol KCl, 1.8 mmol CaCl_2_, 0.1 mmol MgCl_2_, 0.417 mmol NaH_2_PO_4_, 11.9 mmol NaHCO_3_ and 5.5 mmol dextrosis), heated up to 36 °C and aerated mixture of 95% O_2_ and 5% CO_2_. One end of the isolated bowel segment was tied to a holder at the bottom of the bath, while the other was connected to isotonic transducer of F50 physiography (Narco-Bio-System) on which the contractions were registered. 

The adaptation of the preparation took 45 minutes until stable tonus was established (by basal load up to 1.5 g). Following the adaptation, examined substances from the serous side of the bowel were added. In order to avoid the possibility of tolerance creation, the shortest period between additions of two doses was 20 minutes. When injecting high doses, the adaptation periods were extended even up to 30 minutes. The tonus of the isolated bowel segments was measured related to the basal line and presented in millimeters. 

The experimental model consisted of two groups:

In the first experimental series the non-cumulative effect of GBE was examined (0.006–0.06 g/L) on the contractility of the isolated segment of the ileum or the colon.

In the second experimental series on the same bowel excision, first the effect of the growing logarithmic concentration of ACh on the contractility of the longitudinal muscular layer was examined. ACh was applied as follows:

Group A = 6.6 × 10^-9^ mol/L   Group B = 2.2 × 10^-8^ mol/L

Group C = 6.6 × 10^-8^ mol/L   Group D = 2.2 × 10^-7^ mol/L

After at least half-an-hour break, the effects of ACh in GBE were examined again on the same incision (0.06 g/L). Each experiment was repeated at least on eight incisions of certain bowel parts. 

### Plant extract

In the examination the protected German preparation Gingium**®** (Pharmazeutischer Unternehmer XEXAL AG, Hazkki Chen, Germany) was used. The solution was standardized, so that 1 mL contained 40 mg dry extract from ginkgo biloba leaf (35–67:1). Extraction was made by 60% (v/v) acetone. One milliliter preparation contained 8.8–10.8 mg ginkgo flavonol glycoside and 2.0–2.8 mg terpenes with lactone rings (ginkgolides and bilobalides).

### Statistical analysis

Statistical data processing was made on PC by using the statistical package SPSS for Windows, version 8.0. Numerical parameters were presented by one-way analysis of the variants and Student’s t-test. The processed data have been presented in graphs (Harvard Graphics 98). 

## Conclusions

On the basis of the obtained results it may be concluded that GBE reduces spontaneous and ACh-stimulated contractions of the ileum of rabbits which indicates that GBE achieves its effects through cholinergic mechanisms. In the colon GBE reduces spontaneous contractions, but it does not affect on the ACh-stimulated contractions of longitudinal muscles which indicates that the effects are not achieved in cholinergic way.
